# Metabolic engineering of the purine biosynthetic pathway in *Corynebacterium glutamicum* results in increased intracellular pool sizes of IMP and hypoxanthine

**DOI:** 10.1186/1475-2859-11-138

**Published:** 2012-10-24

**Authors:** Susanne Peifer, Tobias Barduhn, Sarah Zimmet, Dietrich A Volmer, Elmar Heinzle, Konstantin Schneider

**Affiliations:** 1 Biochemical Engineering Institute, Saarland University, Campus A1.5, 66123, Saarbrücken, Germany; 2 Institute of Bioanalytical Chemistry, Saarland University, Campus B2.2, 66123, Saarbrücken, Germany

**Keywords:** Purine accumulation, Metabolic engineering, *Corynebacterium glutamicum*, Targeted metabolomics, Metabolic flux analysis

## Abstract

**Background:**

Purine nucleotides exhibit various functions in cellular metabolism. Besides serving as building blocks for nucleic acid synthesis, they participate in signaling pathways and energy metabolism. Further, IMP and GMP represent industrially relevant biotechnological products used as flavor enhancing additives in food industry. Therefore, this work aimed towards the accumulation of IMP applying targeted genetic engineering of *Corynebacterium glutamicum*.

**Results:**

Blocking of the degrading reactions towards AMP and GMP lead to a 45-fold increased intracellular IMP pool of 22 μmol g_CDW_^-1^. Deletion of the *pgi* gene encoding glucose 6-phosphate isomerase in combination with the deactivated AMP and GMP generating reactions, however, resulted in significantly decreased IMP pools (13 μmol g_CDW_^-1^). Targeted metabolite profiling of the purine biosynthetic pathway further revealed a metabolite shift towards the formation of the corresponding nucleobase hypoxanthine (102 μmol g_CDW_^-1^) derived from IMP degradation.

**Conclusions:**

The purine biosynthetic pathway is strongly interconnected with various parts of the central metabolism and therefore tightly controlled. However, deleting degrading reactions from IMP to AMP and GMP significantly increased intracellular IMP levels. Due to the complexity of this pathway further degradation from IMP to the corresponding nucleobase drastically increased suggesting additional targets for future strain optimization.

## Background

The diverse class of purine intermediates comprises phosphorylated nucleotides, nucleosides and nucleobases, exhibiting multiple functions in the cellular system [1]: for example, they serve as transmitters of the genetic information [2], as phosphate group donors, are involved in signal mediation [3] and ensure the energy supply of the living cell [4]. Thus, these intermediates are involved in almost every aspect of cellular metabolism. Furthermore, some of these compounds possess secondary functions as flavor enhancing substances, *i.e.* IMP and GMP [5-7], and act as potential drugs in medical therapy, *e.g.* inosine and purine analogs [8-10]. Due to their immense pharmaceutical and biotechnological potential, these compounds have been successfully synthesized over the past decades via fermentative production employing *Corynebacteria*, especially *Corynebacterium ammoniagenes*[11-13].

**Figure 1:**
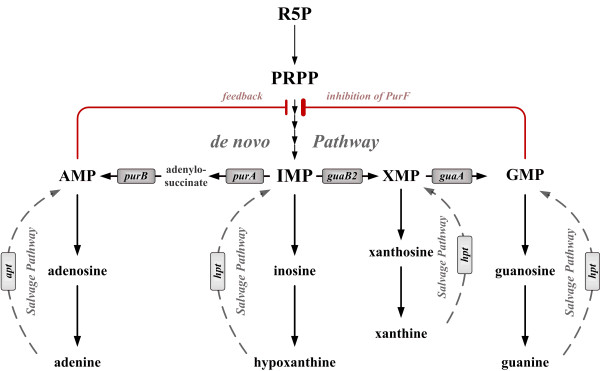
**Metabolic map of the purine biosynthetic pathway. Dashed lines indicate multiple enzymatic steps; boxes denote contributing enzymes of *de novo *biosynthetic pathway (dark gray) and *salvage pathway *(light gray).** Red lines indicate feedback inhibition of the rate controlling step of the purine biosynthetic pathway, regulating the activity of the PRPP amidotransferase by the most stringent inhibitors GMP and AMP based on Zhou et al. [38]. **Abbreviations: R5P**, ribose 5-phosphate; **PRPP**, 5-phosphoribosyl 1-pyrophosphate; **IMP**, inosine 5-monophosphate; **AMP**, adenosine 5-monophosphate; **XMP** xanthosine 5-monophosphate; **GMP**, guanosine 5-monophosphate; ***purA,*** adenylosuccinate synthetase; ***purB***, adenylosuccinate lyase; ***guaB2,*** IMP dehydrogenase; ***guaA,*** GMP synthase; ***apt*,** adenine phosphoribosyltransferase; ***hpt,*** hypoxanthine-guanine phosphoribosyltransferase.

Their *de novo* synthesis, however, is energetically expensive and imbalances in their pool sizes are reported to be directly linked to increased mutation rates [14], which eventually leads to constant pool sizes [2]. These are maintained by a balanced system with two compensatory operating parts: the *de novo* biosynthesis pathway, generating the purine intermediates from central carbon metabolism, and the *salvage pathway*, which can replenish corresponding pools (Figure 1) [6,7,10,15].

*De novo* nucleotide synthesis begins with the activation of the pentose phosphate pathway (PPP) intermediate and direct precursor ribose 5-phosphate [16], which is further converted to IMP in a twelve-step biosynthetic process with concomitant consumption of 10 mol ATP. AMP and GMP are derived from IMP as depicted in Figure 1[1,17]. The *salvage pathway,* however, converts extracellular nucleobases or degraded purine compounds into the corresponding nucleosides and nucleotides [11,18], thereby preventing energetically expensive *de novo* synthesis of these compounds.

Since primary metabolism is designed and optimized to deliver and sustain a more or less consistent distribution of metabolites (described as *network rigidity*[19]) – which can be used as cellular building blocks, redox equivalents, energy and co-factors – any kind of overproduction or accumulation basically targets the deregulation of the underlying metabolism. In the present study, a strategy for increased IMP accumulation was developed by focusing on modifying the general key factors for overproduction: (i) increasing the precursor availability; (ii) de-bottlenecking regulation-restricted branch points, and (iii) deactivating the depleting reactions. The goal of this study was to identify essential nodes of this pathway and to uncover impacts from central and purine metabolism. Applying genetic engineering and in-depth metabolome analysis of the purine pathway intermediates, information of the extremely well-regulated purine pathway should be gathered [1,17,20,21]. However, the underlying mechanisms and the contributions of different branches are not well described yet. In addition, identification of individual regulation factors [22,23] operating at different hierarchic levels [24] is challenging due to the comprehensive connectivity of the metabolic network. The interconnectivity of metabolome and fluxome therefore provides valuable insights into global dynamics [25,26] resulting from single and multiple perturbations in the central and branch metabolism [27].

## Results

### Metabolic snapshots of the purine pathway in wild type *C. glutamicum*

Intracellular metabolite concentrations in *C. glutamicum* were quantified after whole culture extraction using LC-ESI-MS/MS [28]. In order to verify balanced growth conditions [29,30] and stable intracellular metabolite concentrations [31,32], respectively, quantification was performed for two different biomass concentrations, resulting in four biological replicates for each biomass concentration. The corresponding concentrations of nucleotides, nucleosides and nucleobases related to the cell dry weight are listed in Table 1.

**Table 1:** **Concentrations of purine intermediates [μmol g_CDW_^-1^] determined for*C. glutamicum*wild type (ATCC 13032) and site-directed mutants**^**a**^

**Compound**	**Concentration [μmol g_CDW_^-1^]**						
	**ATCC 13032**	***Δpgi***	***purF***^***K348Q***^	***ΔpurA***^***c***^	***ΔguaB2***^***c***^	***ΔpurA ΔguaB2***	***ΔpurA ΔguaB2 purF***^***K348Q***^***Δpgi***
AMP	8.13 ± 0.50	9.07 ± 0.52	10.24 ± 0.78	10.66 (17.93) ± 0.22 (1.18)	10.11 (27.79) ± 0.05 (2.03)	3.69 ± 0.54	3.57 ± 0.20
adenosine	0.10 ± 0.01	0.09 ± 0.01	0.12 ± 0.02	0.13 ± 0.01	0.21 ± 0.01	0.05 ± 0.00	0.15 ± 0.03
adenine	2.63 ± 0.25	3.04 ± 0.11	3.36 ± 0.30	3.04 (5.58) ± 0.38 (0.26)	4.03 (8.32) ± 0.13 (0.53)	^b^	^b^
GMP	2.63 ± 0.16	3.65 ± 0.17	4.75 ± 0.27	9.47 (21.10) ± 0.12 (2.03)	3.92 (2.33) ± 0.19 (0.24)	0.65 ± 0.07	0.51 ± 0.03
guanosine	0.04 ± 0.00	^b^	0.03 ± 0.00	0.03 ± 0.01	0.06 ± 0.01	0.10 ± 0.01	0.23 ± 0.02
guanine	3.27 ± 0.28	3.85 ± 0.17	5.08 ± 0.61	5.07 (10.27) ± 1.45 (0.62)	35.67 (9.38) ± 1.65 (0.64)	^b^	^b^
IMP	0.49 ± 0.06	0.42 ± 0.03	0.55 ± 0.05	30.72 (43.99) ± 0.48 (3.32)	6.39 (37.16) ± 0.21 (3.48)	21.91 ± 0.89	12.88 ± 0.74
inosine	0.13 ± 0.05	0.21 ± 0.04	0.38 ± 0.12	0.28 ± 0.06	0.85 ± 0.06	1.26 ± 0.08	2.53 ± 0.19
hypoxanthine	0.28 ± 0.03	0.29 ± 0.03	0.28 ± 0.03	11.87 (17.92) ± 1.00 (1.26)	3.55 (16.19) ± 0.04 (1.34)	80.65± 3.19	101.53 ± 1.09
XMP	^b^	^b^	^b^	^b^	^n. d.^	^n. d.^	^n. d.^
xanthosine	^b^	^b^	^b^	^b^	^n. d.^	^n. d.^	^n. d.^
xanthine	0.12 ± 0.01	0.22 ± 0.01	0.11 ± 0.00	0.17 ± 0.00	^n. d.^	^n. d.^	^n. d.^

^a^ Concentrations and corresponding standard deviations were determined using 4 biological replicates for each compound and standardized to the cell dry weight.

^b^ Below detection limit.

^c^ Purine intermediate concentrations determined for *C. glutamicum ΔpurA* and *C. glutamicum ΔguaB2* represent maximum values obtained for two biomass concentrations for each strain: numbers on top correspond to the first biomass concentration (1.3 g L^-1^ for *C. glutamicum ΔpurA* and 1.1 g L^-1^ for *C. glutamicum ΔguaB2*) and numbers in brackets for second biomass concentration (1.9 g L^-1^ for *C. glutamicum ΔpurA* and 1.8 g L^-1^ for *C. glutamicum ΔguaB2*).

^n. d.^ not detected.

All compounds except xanthosine and XMP, which were below the detection limit, were quantified with a precision better than 12%. AMP and GMP, as central building constituents for DNA and RNA and co-substrates for various enzyme reactions [2,3], showed the highest concentration ranges of 8.13 and 2.63 μmol g_CDW_^-1^, respectively. Other intermediates varied within a broader concentration range from 0.04 to 3.27 μmol g_CDW_^-1^. The lowest concentrations were detected for nucleosides reaching up to 0.04 μmol g_CDW_^-1^ for guanosine.

Several genetic perturbations aiming at various metabolic key nodes were performed to evaluate the effects and impacts of changing activities of different metabolic branches of the metabolism on the intracellular purine pool sizes.

### Manipulating the main carbon flux distribution

The first implemented modification affected the glucose 6-phosphate branch point and focused on amending the availability of the immediate precursor of IMP, ribose 5-phosphate. The carbon flux distribution was rerouted by deleting the first enzyme of the glycolysis, the glucose 6-phosphate isomerase encoded by the *pgi* gene, resulting in an entry block into glycolysis at that stage and thus restricting the metabolization of glucose 6-phosphate exclusively by the PPP. Growth parameters are depicted in Table 2. The specific growth rate of *C. glutamicum Δpgi* was slightly reduced compared to the wild type strain, as was biomass formation.

**Table 2:** **Growth parameters for *C. glutamicum *ATCC 13032 and site-directed mutants**

**Strain**	**Parameter**		
	**μ**	**Y**_**x/s**_	**q**_**S**_
**ATCC 13032**	0.443 ± 0.004	0.095 ± 0.004	4.67 ± 0.09
***Δpgi***	0.376 ± 0.001	0.090 ± 0.002	4.18 ± 0.02
***purF***^***K348Q***^	0.440 ± 0.008	0.091 ± 0.001	4.82 ± 0.06
***ΔpurA***	0.455 ± 0.006	0.090 ± 0.001	5.05 ± 0.02
***ΔguaB2***	0.320 ± 0.007	0.073 ± 0.004	4.38 ± 0.03
***ΔpurA ΔguaB2***	0.317 ± 0.006	0.075 ± 0.001	4.24 ± 0.01
***ΔpurA ΔguaB2 purF ^K348Q^Δpgi***	0.146 ± 0.003	0.076 ± 0.003	1.92 ± 0.02

Given data represent mean values obtained from two parallel cultivations with corresponding standard deviations: specific growth rate (μ) [h^-1^]; biomass yield (Y_X/S_) [g mmol^-1^] and the specific glucose consumption rate (q_S_) [mmol g^-1^ h^-1^].

Phenotypic analysis was further extended towards the determination of free intracellular purine pool sizes and a metabolic profile based on these analyses was generated (Table 1). Intracellular concentrations related to cell dry weight showed only minor changes compared to wild type *C. glutamicum*. GMP and AMP concentrations were slightly increased in *C. glutamicum Δpgi*, as were amounts of adenine, guanine and inosine. Adenosine and guanosine showed decreased concentrations. IMP and hypoxanthine were not affected significantly.

### Targeted perturbations of the purine biosynthetic pathway

Further genetic manipulations aimed at the purine pathway itself, to estimate immediate effects emanating from this metabolic branch. The first target was the *purF* gene encoding the PRPP amidotransferase. This enzyme is generally regarded to act as key controller in the purine *de novo* synthesis, regulated by a feedback inhibition mainly caused by GMP and AMP [7,33]. An experimentally identified amino acid substitution (K326Q) in the *purF* gene is reported to offset this feedback inhibition by AMP and GMP in *E. coli*[34] and was incorporated into the corresponding position in the *purF* gene of *C. glutamicum*. In order to check for evolutionary conserved coding regions, a sequence alignment covering the complete amino acid sequence was performed [34]. Therefore, six organisms exhibiting a broad phylogenetic distinction, including *Escherichia coli, Corynebacterium glutamicum, Corynebacterium ammoniagenes, Bacillus subtilis, Salmonella typhimurium* and *Saccharomyces cerevisiae,* were analyzed. A conserved region comprising at least ten amino acids was identified for all organisms including the identified location of the amino acid substitution K326Q in *E. coli* described by Zhou et al. [34]. The corresponding substitution K348Q in *C. glutamicum* was inserted into the genomic DNA. Verification of the base exchange was carried out by sequencing.

The amino acid substituent *C. glutamicum purF*K348Q (*purF*^*K348Q*^) showed balanced growth with stable biomass formation and growth rate (Table 2). Intracellular purine intermediate pool sizes remained almost unchanged for adenosine, guanosine and hypoxanthine (Table 1). The most striking changes were observed for intracellular inosine concentration which increased 2.9-fold. Compared to the wild type strain, the IMP concentration, however, showed only a minor change (10% increase). Intracellular concentrations of AMP and its corresponding nucleobase adenine increased by approximately 25% each. GMP and guanine pool sizes in *C. glutamicum purF*^*K348Q*^ increased significantly by 80% and 55%, respectively, compared to the wild type, indicating a global shift towards the main intermediates GMP, AMP and inosine. Direct determination of the *in vitro* activity of PurF following the method of Matsui et al. [35], employing HPLC quantitation of substrate (glutamine) and product (glutamate) concentrations [36] over time in crude cell extracts, resulted in tremendous background activity, not permitting kinetic studies. In addition, heterologous expression using *Escherichia coli* as expression system resulted in enzyme inactivation, probably due to complex posttranslational events. Further studies will however focus on the determination of the *in vitro* activity.

In addition to the deregulation of PurF, further genetic manipulations aimed at IMP degradation, affecting the synthesis of IMP-derived GMP and AMP. The biosynthesis of both intermediates was thus shifted from *de novo* biosynthesis to the purine *salvage pathway*, employing the two enzymes adenosine-phosphoribosyltransferase (APRT) and hypoxanthine-guanine-phosphoribosyltransferase (HGPRT) catalyzing the synthesis of AMP and GMP/IMP via a condensing reaction of adenine and guanine/hypoxanthine with PRPP maintaining a sustained supply of the necessary precursors. These genetic manipulations, however, lead to auxotrophies for adenine and/or guanine [35,37].

As initial strategy, the single gene deletions of the adenylosuccinate synthetase (*purA*) (catalyzing the conversion of IMP to AMP) and of the IMP-dehydrogenase (*guaB2*) (catalyzing the reaction of IMP to GMP) were analyzed. Due to the generated auxotrophies, the medium had to be supplied with adenine and guanine and resulting phenotypes based on intracellular concentrations of the corresponding purine pathway intermediates were analyzed (Table 1).

The metabolic shift from *de novo* purine biosynthesis to the *salvage pathway* did not show any significant changes in the growth rate of the *purA* deletion strain. The *guaB2* deletion strain, however, showed a drastic response after redirecting GMP formation to the *salvage pathway*: biomass yield was reduced by 25% (0.073 g mol^-1^_glucose_) of the wild type yield and the specific growth rate even dropped by 30% of the wild type rate, reaching a stable rate of 0.32 h^-1^, thus indicating a restricted compensation efficiency of the *salvage pathway* concerning the GMP supply. Metabolic phenotyping revealed even more distinct alterations: most strikingly both deletion mutants were characterized by unbalanced intracellular pools sizes, exhibiting transient concentration changes which could not be related to biomass formation (Table 1). Degradation inefficiency of IMP towards the following nucleotides (GMP and AMP) led to maximal intracellular IMP concentrations of 44 μmol g_CDW_^-1^ (*ΔpurA*) and 37 μmol g_CDW_^-1^ (*ΔguaB2*) corresponding to a 90- and 80-fold increase compared to the wild type strain. Hypoxanthine concentrations were affected likewise.

The unbalanced metabolic state observed in *C. glutamicum ΔpurA* and *C. glutamicum ΔguaB2*, was further investigated in the double deletion mutant *C. glutamicum ΔpurA ΔguaB2*, exhibiting auxotrophies for adenine as well as for guanine. Specific growth parameters of the double deletion mutant, *i.e.* biomass yield (0.075 g_CDW_ mmol_glucose_^-1^) and specific growth rate (0.317 h^-1^), were consistent with those obtained for *C. glutamicum* Δ*guaB2*, indicating a mutual growth-related effect. However, the metabolic fingerprint for *C. glutamicum ΔpurA ΔguaB2* exhibited remarkable differences (Table 1): the unbalanced metabolic behavior observed in *C. glutamicum* Δ*purA* and *C. glutamicum* Δ*guaB2* was completely reestablished resulting in constant intracellular purine intermediate concentrations for all substances quantified. The double deletion mutant showed a decreased intracellular IMP pool of 21.9 μmol g_CDW_^-1^ compared to the single gene deletion mutants which corresponds to a 44-fold increase compared to the wild type strain. The IMP reduction was accompanied by a significant increase of the inosine concentration (9.7-fold compared to the wild type strain) and especially hypoxanthine (290-fold compared to the wild type strain), indicating a metabolic shift from main purine intermediates; *i.e*., the nucleotides, to the corresponding degradation products (nucleobases).

### Extended metabolic manipulations – combination of central pathway and branch pathway modifications

Finally, the combined influence of the genetic modifications described above was investigated. The rationally designed mutant *C. glutamicum ΔpurA ΔguaB2 purF*^*K348Q*^*Δpgi* is characterized by the amino acid substitution (*purF*K348Q), supposed to affect the regulatory key enzyme for the *de novo* synthesis. In addition, the mutant exhibits gene deletions for the initiating reaction of glycolysis (*pgi*) and for the converting reactions leading from IMP to the successive purine nucleotides AMP (*purA*) and GMP (*guaB2*).

The biomass yield of 0.076 g_CDW_ mmol_glucose_^-1^ for *C. glutamicum ΔpurA ΔguaB2 purF*^*K348Q*^*Δpgi* was consistent with those obtained for *C. glutamicum ΔpurA ΔguaB2* and *C. glutamicum ΔguaB2*. However, the specific growth rate was reduced to 0.146 h^-1^. In contrast to the two single gene deletion mutants (*ΔpurA* and *ΔguaB2),* the previously observed IMP decline observed in the double deletion mutant *C. glutamicum ΔpurA ΔguaB2* was even more pronounced in the mutant containing the four genetic modifications (Table 1). In conjunction with these findings, an increased IMP degradation towards the corresponding nucleoside inosine and nucleobase hypoxanthine was observed.

### Quantification of intracellular carbon flux distributions

Alterations of *in vivo* carbon flux distributions were investigated in order to assess the impact on the purine intermediate profile. The network contained reactions of glycolysis, TCA cycle, anaplerosis and PPP. Estimation of intracellular flux distributions was carried out based on measurable extracellular rates, *i.e.* glucose consumption, biomass and by-product formation as well as the mass isotopomer data obtained from ^13^C enriched cellular protein. Initial values were statistically varied and multiple parameter estimations were performed to guarantee finding of the global minimum solution.

**Figure 2:**
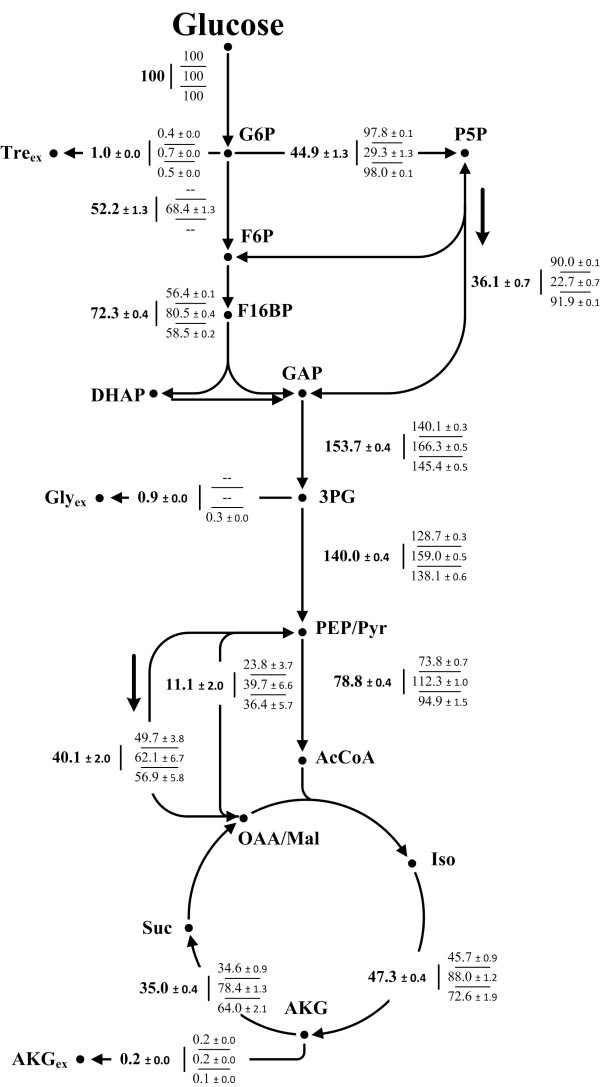
**Intracellular carbon flux distributions with corresponding standard deviations of the central metabolism of the wild type *C. glutamicum *ATCC 13032 (in bold) and mutant strains *C. glutamicum Δpgi, C. glutamicum ΔpurA ΔguaB2 *and *C. glutamicum ΔpurA ΔguaB2 purF ***^***K348Q***^***Δpgi *(displayed in that order from top to bottom).** Net fluxes are given as molar percentage of the mean specific glucose uptake rates (4.67 mmol g^-1^ h^-1^ for *C. glutamicum* ATCC 13032; 4.18 mmol g^-1^ h^-1^ for *C. glutamicum Δpgi*; 4.24 mmol g^-1^ h^-1^ for *C. glutamicum ΔpurA ΔguaB2*; 1.92 mmol g^-1^ h^-1^ for *C. glutamicum ΔpurA ΔguaB2 purF*^*K348Q*^*Δpgi*), which were set to 100%. Anabolic fluxes leading towards biomass formation were not displayed for the benefit of lucidity. Anabolic demands for auxtrophic mutant strains *C. glutamicum ΔpurA ΔguaB2* and *C. glutamicum ΔpurA ΔguaB2 purF*^*K348Q*^*Δpgi* were corrected for the supplementation of adenine and guanine. **Abbreviations: G6P**, glucose 6-phosphate, **P5P**, pentose 5-phosphate; **F6P**, fructose 6-phosphate; **F16BP**, fructose 1,6-bisphosphate; **DHAP**, dihydroxyacetone phosphate; **GAP**, glyceraldehyde 3-phosphate; **3PG**, 3-phosphoglycerate; **PEP**, phosphoenolpyruvate; **Pyr**, pyruvate; **AcCoA**, acetyl-CoA; **Iso**, isocitrate; **AKG**, α-ketoglutarate; **Suc**, succinate; **OAA**, oxaloacetate; **Mal**, malate; **Gly**_**ex**_, extracellular glycine; **Tre**_**ex**_, extracellular trehalose.

**Figure 3:**
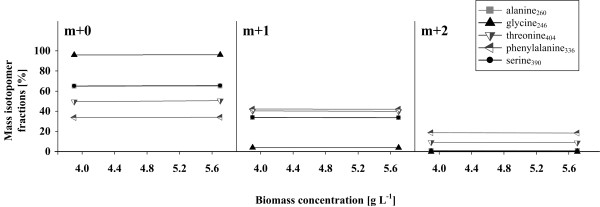
**Mass isotope fractions [%] of amino acid [M-57] fragments derived from different precursor molecules throughout the central carbon metabolism.** Data are shown exemplarily for *C. glutamicum* ATCC 13032.

The metabolic carbon flux distributions determined for the strains *C. glutamicum* ATCC 13032 (wild type) and *C. glutamicum Δpgi, C. glutamicum ΔpurA ΔguaB2* and *C. glutamicum ΔpurA ΔguaB2 purF*^*K348Q*^*Δpgi* are illustrated in Figure 2. Metabolic and isotopic steady state was validated for all strains analyzed by ^13^C MFA. Metabolic steady state was assessed by constant biomass and product yields (Table 2), constant intracellular metabolite concentrations (Table 1) and exponential growth (Table 2). Isotopic steady state was assured comparing mass isotopomer distributions of amino acids for two biomass concentrations. Mass isotopomer fractions of derivatized alanine, glycine, valine, threonine, phenylalanine and serine – determined for *C. glutamicum* ATCC 13032 (wild type) – are highlighted as illustrative examples in Figure 3. Isotopic steady states for *C. glutamicum Δpgi, C. glutamicum ΔpurA ΔguaB2* and *C. glutamicum ΔpurA ΔguaB2 purF*^*K348Q*^*Δpgi* were assured likewise and exhibited comparable results (data not shown).

Deletion of the *pgi* gene strongly affected the carbon flux distribution and caused a complete carbon redirection towards the PPP. The reflux of ribose 5-phosphate via the non-oxidative part of the PPP entering glycolysis was almost tripled. Activity of the TCA cycle was only slightly affected, showing a small reduction from 47.3% in the wild type strain to 45.7% in the *pgi* deletion mutant.

In contrast, the double deletion mutant *C. glutamicum ΔpurA ΔguaB2,* possessing an intact *pgi* gene, exhibited the lowest flux through the oxidative part of the PPP (29.3%), which almost exclusively accounted for biomass formation. The relative reflux into glycolysis was less than 10%. TCA cycle activity was increased to 88%. The strain *C. glutamicum ΔpurA ΔguaB2 purF*^*K348Q*^*Δpgi* showed a carbon flux redirection from glycolysis towards the PPP (98.0%) caused by the *pgi* gene deletion.

### Perturbation-caused alterations of the NADH metabolism

**Figure 4:**
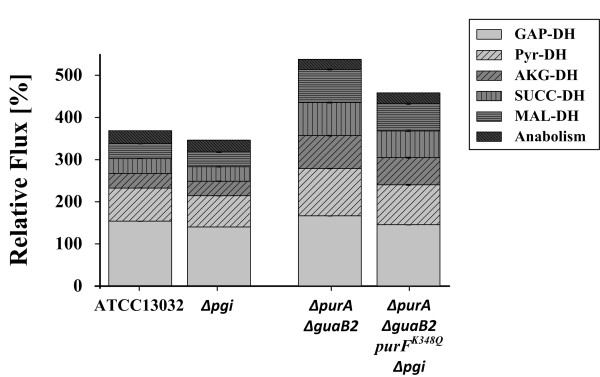
**XADH formation for *C. glutamicum *ATCC 13032, *C. glutamicum Δpgi, C. glutamicum ΔpurA ΔguaB2 *and *C. glutamicum ΔpurA ΔguaB2 purF^K348Q ^Δpgi *.** Glyceraldehyde 3-phosphate dehydrogenase (GAP-DH), pyruvate dehydrogenase (Pyr-DH), α-ketoglutarate dehydrogenase (AKG-DH) and malate dehydrogenase (MAL-DH) were considered as NADH supplying reactions and succinate dehydrogenase (SUCC-DH) as FADH_2_ supplying reaction. Anabolically derived NADH was considered with 3.2 mmol g_CDW_^-1^[40]

The inserted genetic changes also altered the redox metabolism, especially regarding the NADH/FADH_2_ (XADH) supply. A surplus was detected in all strains investigated, which could be used for ATP generation by oxidative phosphorylation. However, the relative NADH production exhibited significant differences among the modified strains (Figure 4): *C. glutamicum* ATCC 13032 produced a XADH surplus of 369% which decreased to 346% in the *pgi* deletion mutant. An increased surplus of 538% was determined for *C. glutamicum ΔpurA ΔguaB2*. This difference can be ascribed to the low activity of the PPP (29%) and the contrariwise high TCA cycle activity (88%). In comparison to *C. glutamicum ΔpurA ΔguaB2, C. glutamicum ΔpurA ΔguaB2 purF*^*K348Q*^*Δpgi* exhibited a decreased XADH surplus of 458.2%.

The consequences of the unequal NADH/FADH_2_ supply are discussed in more detail in *“Cluster analysis” (*see section *Discussion).*

## Discussion

### Rerouting metabolic fluxes – contributions of individual pathway branches with respect to purine accumulation

#### Targeted perturbations in the purine biosynthetic pathway

Imbalances in free intracellular purine pools are reported to be mutagenic and genotoxic [20,21]. Thus a prerequisite for balanced cellular growth is to maintain the intracellular pool sizes, which resulted in efficient regulatory mechanisms [1,16,17,33,35,38]. This pool stability is on the one hand sustained by an interaction of *de novo* purine synthesis and the *salvage pathway*, ensuring a constant supply of purine nucleotides required for cellular growth [16]. On the other hand, increasing concentrations perturbing the system can easily be broken down via the present degrading and converting reactions.

In case of the *purA* gene deletion mutant (*C. glutamicum ΔpurA)*, extracellular supplementation with adenine completely restored cellular growth, resulting in comparable specific growth rates of the wild type (0.443 h^-1^) and the *C. glutamicum ΔpurA* strain (0.455 h^-1^), indicating a thorough compensatory effect by the *salvage pathway*.

On the contrary, extracellular guanine complementation was not sufficient to restore wild type growth behavior in the *guaB2* gene deletion mutant (*C. glutamicum ΔguaB2)*: specific growth rate as well as biomass yield were reduced by 25%, each compared to the wild type strain (Table 2). This can either be explained by insufficient uptake or converting systems for guanine. The latter is catalyzed by the *salvage pathway*. The observed phenotypic consequences reemerged in every genotype comprising this genetic perturbation (*C. glutamicum ΔguaB2* and *C. glutamicum ΔpurA ΔguaB2),* no matter if single or multiple genes were deleted. The attribution of this fundamental *salvage* branch restriction regarding uptake or conversion could not be answered definitely in this study. Decreasing intracellular guanine concentrations in *C. glutamicum ΔguaB2,* however indicate an uptake insufficiency (and not a conversion insufficiency!). Decreasing metabolite concentrations are caused by increased consuming reactions (in this case by the *salvage pathway*).

In contrast, the *purA* gene deletion mutant *C. glutamicum ΔpurA* exhibited increasing adenine concentrations, supporting a sufficient adenine uptake, even exceeding converting reactions by the *salvage pathway*. This theory is additionally supported by the reduced guanine uptake (125 μmol g_CDW_^-1^) compared to the adenine uptake (243 μmol g_CDW_^-1^), indicating basic differences for both intermediates.

Regarding intracellular pool sizes, the single gene deletion mutants *C. glutamicum ΔpurA* and *C. glutamicum ΔguaB2* were found to exhibit the most promising rises in intracellular IMP concentration with maximal IMP pool sizes of 44.0 and 37.2 μmol g_CDW_^-1^, respectively. These associated transient concentration changes showed no relation to biomass formation and resulted in unstable and thus unrepresentative results. This phenomenon may at least partially be associated with direct changes in the GMP and AMP pool sizes (Table 1). These two compounds are reported to act as the most stringent regulators for the purine biosynthetic pathway by a feedback-mediated inhibition of the PurF enzyme [16]. With both intermediates operating as regulators, it is not surprising that the individual gene deletions cause spreading influences on the whole branching pathway.

#### Targeted perturbation in the central carbon metabolism

Increasing the precursor supply via the oxidative branch of the PPP seemed to be a promising route to boost intracellular purine levels. This strategy has already been successfully applied for tryptophan production in *E. coli*[39] and lysine production in *C. glutamicum*[40,41]*,* illustrating positive correlations between enhanced productivity and increased PPP activities [42].

Thus, the genetic modification (deletion of the *pgi* gene) was implemented into the genome. The resulting affects on the purine intermediate pool sizes (Table 1) however, were rather limited compared to those observed for *purA* and *guaB2* gene deletion strains, indicating the strong regulation and rigidity of this pathway.

### Effects of combined perturbations on intracellular purine pool sizes

Strains used for industrial manufacturing of purine intermediates are usually characterized by an adenine auxotrophy [35,37,43-45]. This perturbation seems to be fundamental, revealing the importance of an IMP conversion block for the accumulation of this intermediate metabolite [46]. The extracellular supplementation with adenine, however, is linked to a repression of transcription initiation [37] of the genes of the *pur* operon [37] via the depletion of PRPP as described for *B. subtilis*. In order to counterbalance this phenomenon, the carbon flux was rerouted by introducing the *pgi* gene deletion, aiming at an increased PRPP supply (Figure 2).

The carbon flux was successfully rerouted. Intracellular purine levels though further shifted from nucleotides towards their degradation products, mainly inosine and hypoxanthine (Table 1).

### Cluster analysis

**Figure 5:**
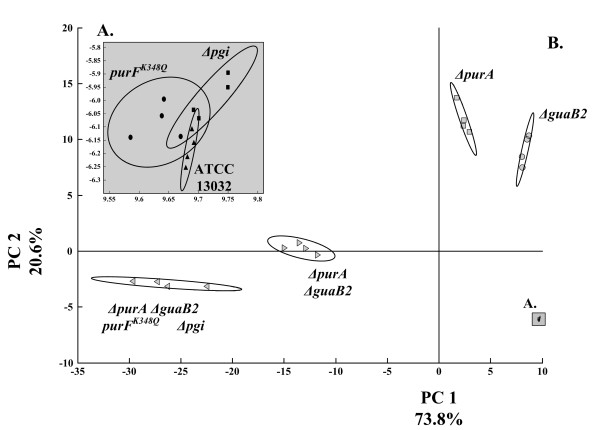
**PCA score plots of principal components 1 and 2 for *C. glutamicum *ATCC 13032, *C. glutamicum Δpgi, C. glutamicum purF^K348Q^, C. glutamicum ΔpurA, C. glutamicum ΔguaB2, C. glutamicum ΔpurA ΔguaB2 *and *C. glutamicum ΔpurA ΔguaB2 purF^K348Q ^Δpgi.*** The metabolite profiling was performed using cell extracts of exponentially growing cultures (n = 4 for each strain). The respective seven strains are shown with corresponding 95% confidence intervals. Data were pareto scaled. The gray insert depicts the analysis of *C. glutamicum* ATCC 13032, *C. glutamicum Δpgi* and *C. glutamicum purF*^*K348Q*^ (zoomed from the right lower corner)*.* For *C. glutamicum ΔpurA* and *C. glutamicum ΔguaB2*, data obtained for phase 2 were considered (see Table 1).

In order to analyze correlations between the different manipulated pathway branches, *principal component analysis* (PCA) was performed. The result of the cluster analysis is illustrated in Figure 5. Pareto-scaled concentrations of purine pathway intermediates were used for the PCA, resulting in the global purine metabolite phenotype for each mutant.

**Figure 6:**
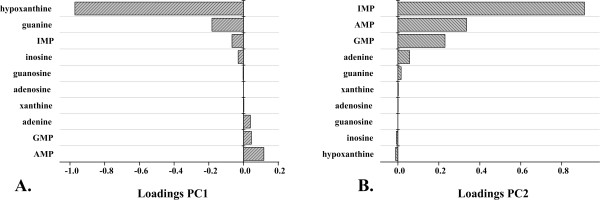
**Loading coefficients for the first (A.) and second (B.) principal component obtained from multivariate analysis (Figure**5**).**

The first two components contributed to more than 90% of the total variance. As shown in the score plot, a distinct separation pattern defining two groups could be derived (Figure 5). The first group (A.), comprising the wild type strain, the *pgi* deletion strain and the *purF* substituent, did not show any significant differences concerning intracellular purine intermediate pool sizes. As discussed earlier, only minor changes with respect to intracellular concentration of purine pathway intermediates were caused by these genetic manipulations, which in direct comparison to the second group (B.) were quite consistent. The second group (B.), however, resulted in a broader clustering pattern comprising intrinsic dissimilarities. As shown by loading coefficients (Figure 6), the separation was mainly caused by the component IMP and its degradation product hypoxanthine, reflecting the observed shift from nucleotide accumulation towards increased degradation.

The disruption of critical nodes in the purine pathway itself, *i.e.* the conversion of IMP to AMP and GMP, provided the required fundamental basis for far-reaching effects when combined with additional modifications as the *pgi* gene deletion.

Solely based on the metabolome data, no clear link between the additional genetic manipulation of the *pgi* deletion and the increased degradation shift could be concluded. Therefore, the research was extended by an in-depth fluxome analysis to capture integrative effects of fluxome and metabolome.

### Connectivity of targeted metabolome and fluxome analysis

In the context of purine accumulation, alterations in the PPP-TCA cycle ratio were of special interest, as their relative activities and especially the flux partitioning into these two main branches, mainly dictate the XADH (NADH+FADH_2_) supply and thus ATP formation in the respiratory chain [47]. The exclusive metabolization of glucose 6-phosphate via the PPP caused a major carbon flux redistribution in *C. glutamicum ΔpurA ΔguaB2 purF*^*K348Q*^*Δpgi*, exhibiting an adverse effect on XADH formation compared to *C. glutamicum ΔpurA ΔguaB2* (Figure 4) and initiating a shift in the metabolic profile towards inosine and especially hypoxanthine formation. Since most XADH is generated in the TCA cycle, high PPP activities inevitable lead to an increased CO_2_ production via a decarboxylation reaction in the oxidative part of the PPP and thus to a reduced carbon availability for other metabolic branches, as seen for *C. glutamicum ΔpurA ΔguaB2 purF*^*K348Q*^*Δpgi* (Figure 2)*.* Within the scope of the tightly regulated purine biosynthesis, Noguchi et al. [47] presumed a connection between a declining ATP supply and an increasing degrading efficiency within this pathway. The results obtained in this study are consistent with those obtained previously [47].

This suggests further metabolic engineering targets to increase intracellular IMP levels focusing on IMP degrading reactions.

## Conclusions

In the present study, we performed an in-depth metabolic profiling of the purine biosynthetic pathway in *C. glutamicum*. Site-directed perturbations on the genetic level with subsequent studies of growth behavior and targeted metabolome analysis revealed a tight regulation of the underlying pathway with strong additional effects on the carbon flux distributions in the main metabolism as determined for *C. glutamicum ΔpurA ΔguaB2* and *C. glutamicum ΔpurA ΔguaB2 purF*^*K348Q*^*Δpgi.*

Our results underline the importance of combined analyses employing different hierarchical levels such as fluxome and metabolome to reveal trends which indicate active regulation mechanism. The regulatory processes maintaining balanced intracellular concentrations have not been fully discovered yet and necessitate further investigation. Complex future studies will thus focus on the redox and energy metabolism, which were beyond the scope of the present analyses. In addition, it might be interesting to further analyze the metabolic instabilities – detected for *C. glutamicum ΔpurA* and *C. glutamicum ΔguaB2.* These instabilities were caused by the alteration of the complex regulatory structure of this pathway branch, opening up a way for in-depth surveys of this central and essential pathway in prokaryotes as well as in eukaryotes.

In order to achieve an understanding of the global cellular response, individual parts of the underlying network*, i.e*. precursor and cofactor supply, energy and redox metabolism, should not be assumed as independent, individual but interconnected units, linked by various key metabolites such as ATP or NAD(P)H.

## Methods

### Organisms and plasmids

Wild type *C. glutamicum* ATCC 13032 was obtained from the American Type Culture Collection (Manassas, VA, USA) and used as parent strain for further genetic modifications. *E. coli* DH5α was utilized as host strain for cloning and plasmid construction. *E. coli* NM522^pTC^ served as host for plasmid amplification and coryne-specific methylation. The pTC plasmid, harboring an origin of replication for *E. coli* (*ori*), a tetracycline resistance (Tet^R^) as selection marker and the DNA-methyltransferase gene from *C. glutamicum*, was co-expressed in *E. coli* NM522^pTC^ to transfer the methylation pattern of *C. glutamicum* to the integrative plasmids. The vector pClik was used for integrative genetic modifications. The modifications constructed in this study comprised single gene deletions (*ΔpurA, ΔguaB2* and *Δpgi*), an allelic replacement of the *purF* gene encoding amidophosphoribosyltranferase with a *purF*K348Q gene (*purF*^*K348Q*^) and multiple gene deletions (*ΔpurA ΔguaB* and *ΔpurA ΔguaB2 purF*^*K348Q*^*Δpgi*). The site-directed mutants and corresponding genetic modifications are summarized in Table 3. Allelic replacement of the modified *purF* gene was carried out by first deleting the native gene followed by a replacement step with the manipulated gene containing the point mutation. Primer sequences used for construction and verification are listed in Table 4.

**Table 3:** **Genetically modified organisms constructed in the present study: Mutant strains of *C. glutamicum *based on the wild type strain (ATCC 13032) were constructed by site-directed mutagenesis**

**Strain**	**Genotype**	**Reference**
*C. glutamicum* ATCC 13032	wild type	American Type Culture Collection
*C. glutamicum Δpgi*	deletion of the *pgi* gene (NCgl0817)	This work
*C. glutamicum ΔpurA*	deletion of the *purA* gene (NCgl2669)	This work
*C. glutamicum ΔguaB2*	deletion of the *guaB2* gene (NCgl0578)	This work
*C. glutamicum purF*^*K348Q*^	Nucleotide exchange K348Q in the *purF* gene (NCgl2495)	This work
*C. glutamicum ΔpurA ΔguaB2*	double deletion of *purA* and *guaB2* genes	This work
*C. glutamicum ΔpurA ΔguaB2 purF*^*348*^*Δpgi*	deletion of *purA*, *guaB2* and *pgi* genes and allelic amino acid substitution K348Q in the *purF* gene	This work
*E. coli* DH5α	F^−^endA1, hsdR17(rk^−^mk^+^) supE44, thi-1λ^−^ recAI gyrA96 relA1 ^Φ^80ΔlacAm15	[48]
*E. coli* NM522^pT*C*^	*supE thi-1* Δ(*lac-proAB*) Δ*(mcrB-hsdSM)5 (r*K^−^*m*K^+^) [F′ *proAB lacI*q*ZΔM15*]	Stratagene

*E. coli* strains were employed for plasmid construction.

**Table 4:** Mutant genotypes and corresponding site-specific primer sequences (F, forward; R, reverse) used for verification^a^

**Mutant genotype**	**Primer**	**Primer sequence**
*Δpgi*	*Δpgi-*F	5′*-*GATCACGCGTATCCCTTCTCCGGCATC*-*3′
	*Δpgi-*R	5′*-*GATCTCTAGATCCAGCGACACGAATAATC*-*3′
*ΔpurA*	*ΔpurA-*F	5′*-*GATCTCTAGAATGGATCGGATGTGACG*-*3′
	*ΔpurA-*R	5′*-*GATCACGCGTCAATCGGTCAACCTGGT*-*3′
*ΔguaB2*	*ΔguaB2-*F	5′*-*GATCACGCGTGAGTTTCTACCGGAGGA*-*3′
	*ΔguaB2-*R	5′*-*GATCTCTAGATCAGACTGGAAGTAACG*-*3′
*purF*^*K348Q*^	*purF*^*K348Q*^*-*F	5′*-*GATCACGCGTGATTGCGGACTGGTTAC*-*3′
	*purF*^*K348Q*^*-*R	5′*-*GATCTCTAGACTCCTGCTGCTGCGTATG*-*3′
	K348Q*-*1	5′*-*GGCCAAGGCATGGTCCAGAACGCCTACGTTGGC*-*3′
	K348Q*-*2	5′*-*GCCAACGTAGGCGTTCTGGACCATGCCTTGGCC*-*3′

^a^ For the single amino acid substitution in *purF*^*K348Q*^, sequences for fusion primers (K348Q-1, K348Q-2) containing the point mutation (underlined) are denoted.

### DNA preparation and recombinant DNA modifications

Oligonucleotide synthesis was performed by Sigma-Aldrich (Steinheim, Germany) and DNA sequencing was carried out by GATC (Konstanz, Germany). Plasmid construction and DNA purification were performed using standard techniques. Preparation of genomic DNA from *C. glutamicum* was carried out with the MasterPure^TM^ Gram Positive DNA Purification Kit (Biozym Scientific GmbH, Germany). PCR reactions were performed in a TGradient-Cycler (Whatman–Biometra^R^) with Phusion polymerase (Biozym) for insert construction and JumpStart REDTaq Ready Mix (Sigma-Aldrich) for strain verification. PCR products were purified using NucleoSpin® Extract II Kit (Macherey-Nagel, Düren, Germany) and plasmids using HiYield® Plasmid Mini Kit (SLG, Gauting, Germany). Ligations were performed with Fast-Link^TM^ DNA Ligation Kit (Epicentre Biotechnologies, Madison, WI, USA). FastDigest® restriction enzymes were obtained from Fermentas (St. Leon-Rot, Germany) as well as 10 mM dNTP Mix and 1-kb DNA O’GeneRuler^TM^.

### Transformation

Precultures used for transformation of *C. glutamicum*, were grown on BHI^++^ medium (10 mL in 100 mL baffled shake flasks) and incubated for 12 h at 30°C. The main culture in BHI^++^ medium (80 mL in 1000 mL baffled shake flasks) was inoculated from exponentially growing precultures to an optical density OD_660nm_ of 0.2. 400 mg isonicotinic acid hydrazide, 2.5 g glycine and 0.1 mL Tween 80 were dissolved in 20 mL H_2_O, sterilized by filtration and added to the main culture at an OD_660nm_ of 0.6. Cells were further incubated until an OD_660nm_ of 0.8 was reached and harvested by centrifugation (7 min, 4000 *g*, 4°C, Laborfuge 400R, Heraeus, Hanau, Germany). Cells were washed three times with pre-cooled wash buffer (10% glycerine, 8 mM tris hydrochloride, pH 7.4) and once with 10% glycerine. After the second resuspension in 6 mL 10% glycerine, 200 μL cells were subsequently used for transformation. Electroporation was performed using a BioRad Gene Pulser II (Hercules, California, USA) with the following parameters: 25 μF, 600 Ω and 2.5 kV. Cells were transferred into a 2 mL Eppendorf tube, mixed with 1 mL BHIS medium, followed by a heat shock for 5 min at 46°C. Regeneration was performed for 90 min at 30°C to allow recovery of the cells, which were then spread on BHIS^Kan^ agar plates and incubated for 4 days.

Preparation of heat shock competent *E. coli* cells was performed as described by Inoue et al. [49]. Transformation of *E. coli* DH5α and NM522^pTC^ with the integrative pClik-derivates was performed by heat shock [49]. Briefly, cells were incubated on ice for 30 min, heated at 45°C for 45 s and again cooled on ice for 2 min. Regeneration was performed for 1 h at 37°C after 900 μL SOC medium were added.

### Targeted mutant generation

Site-directed mutations were introduced using the integrative vector pClik, which lacks an *ori* for *C. glutamicum*. Selection on Kan^R^ as resistance marker yielded transformed cells with genome-integrated plasmid DNA. The insertion of the plasmids thereby results from a single crossover recombinant event. The second homologous recombination event, generated by the *sacB* positive selection system [50], causes the deletion of the *sacB* gene and therefore allows growth on corresponding selective agar plates. Due to the *sacB* associated additional deletion of the native or the modified gene sequence, transformants either contain the wild type gene sequence or exhibit the targeted mutation. Kan^R^-nonresistant and sucrose-insensitive clones were further verified by allelic-specific PCR for the presence of the targeted gene sequence. The corresponding gene sequence for a nucleotide exchange, *i.e. purF*^*K348Q*^, was first deleted and then replaced.

### Chemicals

Yeats extract, beef extract, polypeptone, casamino acids, and brain heart infusion (BHI) were purchased from Difco Laboratories (Detroit, USA). 99% enriched glucose, *i.e*. [1-^13^C] and [1,2-^13^C_2_], were obtained from Cambridge Isotope Laboratories (Andover, MA., USA). All other chemicals and reagents of analytical grade were purchased from Sigma-Aldrich, Merck (Darmstadt, Germany), Fluka (Buchs, Switzerland) or Roth (Karlsruhe, Germany).

### Growth Media

The first precultures were grown on complex media (pH 6.8) containing per liter: 10.0 g glucose, 2.5 g NaCl, 2.0 g urea, 5.0 g yeast extract, 5.0 g beef extract, 5.0 g polypeptone and 20.0 g casamino acids. For agar plates, the complex medium was supplemented with 20.0 g L^-1^ agar. Second precultures and main cultures were grown on minimal medium (pH 7.2) containing per liter: 15.0 g glucose, 4.0 g KH_2_PO_4_, 16.0 g Na_2_HPO_4_, 500 mg MgCl_2_ · 6H_2_O, 300 mg 3,4-dihydroxibenzoic acid, 100 mg CaCl_2_ · 2H_2_O, 100 μg cyanocobalamin, 750 μg thiamine, 4 μg pyridoxal phosphate, 100 μg biotin, 400 μg calcium pantothenate, 2 μg folic acid, 400 μg nicotinic acid, 200 μg 4-aminobenzoic acid, 400 μg pyridoxine · HCl, 2 mg inositol, 10 mg FeCl_2_ · 4H_2_O, 1 mg ZnCl_2_, 100 μg CuCl_2_, 20 μg NiCl_2_ · 6H_2_O, 20 μg Na_2_MoO_4_ · 2H_2_O, 500 μg boric acid, 100 μg KI, 100 μg CoCl_2_ · 6H_2_O and 10 mg MnCl_2_ · 4H_2_O. For tracer studies, naturally labeled glucose was replaced by 99% ^13^C enriched [1-^13^C] or [1,2-^13^C] glucose. Mutants of *C. glutamicum* exhibiting deletions of adenylosuccinate synthetase (*purA*) or IMP dehydrogenase (*guaB2*) were grown on minimal media additionally supplemented with 200 mg L^-1^ adenine sulfate or 80 mg L^-1^ guanine hydrochloride, due to the introduced auxotrophies.

Media used for transformation experiments of *C. glutamicum* contained per liter: 37 g BHI, 26.4 g (NH_4_)_2_SO_4_ and 40 g glucose for BHI^++^ and 37 g BHI and 46 g sorbitol for BHIS medium.

### Cultivations

Cultivations were carried out in baffled shake flasks on an orbital shaker (Multitron 2, Infors AG, Bottmingen, Switzerland) at 230 rpm and 30°C. First precultures were grown on complex medium (25 mL medium in 250 mL baffled flask) for 8 h and used for inoculation of the second precultures (25 mL medium in 250 mL baffled flask). Second precultures and main cultures were performed in minimal medium. Main cultivations were performed in duplicates in minimal medium using 500 mL baffled shake flasks with 50 mL medium. For tracer experiments, which were conducted under the same conditions as main cultivations, naturally labeled glucose was replaced by [1-^13^C] glucose or [1,2-^13^C_2_] glucose for analysis of glucose 6-phosphate isomerase (*pgi*) deletion mutants and the volume was adjusted to 5 mL in 50 mL baffled shake flasks.

### Substrate and product analysis

Biomass concentration was determined by measuring the optical density of the culture at 660 nm (OD_660nm_) using a photometer (Marsha Pharmacia Biotech, Freiburg, Germany) which was gravimetrically correlated with the cell dry weight The correlation factor was determined as 0.414 g_CDW_ OD_660nm_^-1^. Extracellular substrates and secreted products were analyzed after 3 min centrifugation at 16,000 *g*. Quantification of glucose, α-ketoglutarate and trehalose was performed in diluted supernatants by HPLC (Kontron Instruments, Neufahrn, Germany). Separation was performed using an Aminex HPX-87H Bio-Rad column (300 x 7.8 mm; Hercules, USA), at 55°C with a constant flow rate of 0.8 mL min^-1^ (7 mM H_2_SO_4_ as mobile phase). Refraction index (RI) detection was used for quantification of sugars, and UV detection at 218 nm for organic acids and at 254 nm for quantification of supplemented adenine and guanine. Determination of amino acids was performed as described previously [36]. Extracellular concentrations of adenine and guanine were determined by HPLC (Agilent 1290 Infinity; Agilent Technologies, Waldbronn, Germany) using a Phenomenex (Aschaffenburg, Germany) reversed-phase C18-column (Gemini 5u 110Å, 150 x 4.6 mm). Separations were carried out at 10°C with a constant flow of 0.8 mL min^-1^. Gradient elution was performed using 5 mM ammonium formate solution at pH 5.8 (eluent A) and acetonitrile:methanol:water mixture (45:45:10, eluent B) applying the following gradient: an initial isocratic step with 0% B (3.5 min) was followed by an increase to 30% within 10 min and to 100% within 1 min. The flushing step at 100% B was performed for 5 min. The column was reconditioned at 0% B for another 5 min.

### Analysis of intracellular metabolites

Extraction and quantification of intracellular purine intermediates, *i.e*. nucleotides, nucleosides and nucleobases, by LC-ESI-MS/MS was performed as described previously [28].

### Mass spectrometric ^13^C-labeling analysis

The mass isotopomer distributions of amino acids derived from cellular protein and of trehalose secreted during cultivation were determined by mass spectrometry–gas chromatography. Sample preparation and instrument settings were performed as described previously [41,51,52]. In order to ensure isotopic steady state, labeling patterns of amino acids and trehalose were determined for two biomass concentrations during mid-exponential growth. Metabolic (pseudo-) steady state was assessed by constant yields, uptake and secretion rates and constant intracellular pool sizes of purine intermediates.

### Fluxome analysis

Calculation of the *in vivo* metabolic fluxes of the *C. glutamicum* strains was carried out by fitting simulated mass isotopomer distributions to experimentally determined labeling patterns of amino acids derived from cellular protein and trehalose secreted during cultivation. The calculations were based on the approach of Yang et al. [53] implemented in Matlab (version 2008b, Mathworks Inc., Nattick, USA) using metabolite and isotopomer balancing. Anabolic demands for biomass synthesis and extracellular production rates ware additionally considered [54] and implemented in the metabolic network. Media supplementation with adenine and guanine – required by strains comprising adenylosuccinate synthetase and/or IMP dehydrogenase deletions – led to reduced anabolic demands for DNA and RNA syntheses. The corresponding reduced anabolic demand for 3-phosphoglycerate was considered. In addition, increased ribose 5-phosphate requirements due to increased intracellular pool sizes of IMP, determined for *C. glutamicum ΔpurA ΔguaB2 purF*^*K348Q*^*Δpgi* and *C. glutamicum ΔpurA ΔguaB2,* were considered likewise.

Measured GC-MS data were corrected for naturally occurring isotopes in the corresponding amino acid fragments and derivatization reagent respectively [55,56]. A Monte Carlo approach with 100 independent runs was employed for the statistical analysis [57].

### Data analysis

Mean concentration values for purine intermediates were calculated from four biological replicates for each strain for two biomass concentrations and corrected using ^13^C-labeled cell extract as internal standard as described previously [28]. The metabolomics-based phenotype profiling was extended by principal component analysis (PCA) applying pareto scaling of the data. Data sets were compressed by reduction of dimension numbers and the metabolomic patterns were highlighted. PCA was performed using Matlab R2007b (The Math Works, Inc., Boston, USA) software.

## Competing interests

The authors declare that they have no competing interests.

## Authors’ contributions

SP, KS, EH were involved in conception and design of experiments. SP, TB, SZ performed the targeted strain engineering and cultivation experiments. SP carried out analytical quantitation supported by DAV and KS. SP, KS, DAV and EH were involved in manuscript preparation. KS and SP were involved in data analysis and interpretation. KS drafted and revised the manuscript. All authors read and approved the final manuscript.
